# Chronic Anti-HMG-CoA Reductase Positive Necrotizing Myositis With Remote Exposure to Statins

**DOI:** 10.7759/cureus.40552

**Published:** 2023-06-17

**Authors:** Tooba Salar, Massiel Jimenez, Mazin Hameed, Anthony Ocon

**Affiliations:** 1 Department of Internal Medicine, Rochester Regional Health - Unity Hospital, Rochester, USA; 2 Department of Rheumatology, University of Rochester Medical Center, Rochester, USA; 3 Division of Allergy, Immunology, and Rheumatology, Rochester Regional Health - Unity Hospital, Rochester, USA

**Keywords:** necrotizing myositis, anti-hmgcr, myopathy, myositis, statins

## Abstract

The use of statins may be associated with muscle-related side effects ranging from myalgia to rhabdomyolysis. A rare adverse effect is statin-induced anti-hydroxy-3-methyl-glutaryl-coenzyme A reductase (anti-HMGCR) necrotizing myositis, which may develop after exposure to statins due to autoantibodies against HMG-Co-A reductase. We present the case of a 76-year-old male who developed progressive muscle weakness three years after exposure to statins. He had significantly elevated creatine kinase (CK) levels, despite the discontinuation of statins three years prior. He complained of generalized muscle weakness, and examination revealed reduced strength, especially in the proximal musculature. MRI revealed inflammatory myositis of the medial and posterior compartments of bilateral thighs. Autoimmune workup was positive for anti-HMG-CoA reductase antibodies. Muscle biopsy showed endomysial inflammation with fibrosis and fat replacement, suggesting chronic but active myositis. A diagnosis of chronic anti-HMGCR necrotizing myositis was made. The patient was started on oral prednisone and methotrexate with improvement in symptoms and CK levels. This case highlights a chronic form of a rare cause of myositis that may be a challenge to diagnose given the remote exposure to statins.

## Introduction

Statins are commonly prescribed for cardiovascular risk reduction and management of hyperlipidemia, especially in the outpatient setting. Reported side effects include myalgia and rhabdomyolysis which are the most common reasons for discontinuation [1,2]. A rare adverse effect is necrotizing immune-mediated myositis, which may persist even after cessation of statins and poses a challenge in diagnosis and treatment [3].

## Case presentation

A 76-year-old African American male was admitted for fatigue, myalgia, and proximal muscle weakness with a creatine kinase (CK) of 13,546 U/L. The patient had a similar presentation three years prior to the current presentation. During that time, he had initiated the use of atorvastatin for four months and then developed generalized myalgia and subjective lower extremity weakness but had an intact ability to walk, with a CK of 12,433 U/L. The patient described improvement in symptoms upon discontinuation of atorvastatin. At that time, his CK level was monitored with a decreased level to 1025 U/L at one month and then to 382 U/L at three months post-discontinuation. Given his moderate improvement, no further autoimmune workup was done. Since then, he had not been on any additional statin or dyslipidemia medications. However, during the current admission, the patient described a history of ongoing intermittent muscle weakness and myalgia over the past three years. Previous elevations in CK were noted over the past two years without an attributable cause.

He denied hematuria, flank pain, chest pain, history of trauma, prolonged immobility, strenuous exercise, illicit drug use, or nutritional supplement use. He denied dysphagia, dyspnea, cough, rash, arthritis, or Raynaud’s phenomenon. He did not have a prior history of thyroid disease, coronary artery disease, liver disease, or kidney disease. Initial vital signs demonstrated a blood pressure of 135/65 mmHg, heart rate of 78 beats per minute, respiratory rate of 18 per minute, and oxygen saturation of 99% on room air. Neuromuscular examination revealed 4/5 strength of the deltoids and triceps with 5/5 strength in the biceps, forearm flexor and extensor muscles, and intrinsic finger muscles, and 3/5 strength of the hip flexors, quadriceps, lower extremity abductor and adductor muscles, with 4/5 of the hamstring muscles, and 5/5 of the ankle flexor and extensor muscles and intrinsic muscles of his feet. He did exhibit mild diffuse tenderness in his quadriceps, adductor, and hamstring muscles. Sensory examination to light touch was otherwise normal. Cranial nerve examination was normal, and he did not have any bulbar symptoms. The triceps reflex was one of four, and the quadriceps knee jerk reflex was one of four. Other reflexes, including ankle jerk and Babinski, were normal. Gait examination was limited as the patient did not feel strong enough to ambulate independently without falling. The rest of the physical exam was normal.

The patient’s past medical history included hyperlipidemia treated with atorvastatin 20 mg that was last taken over three years ago before presentation, rectal adenocarcinoma diagnosed 2.5 years prior to the presentation but after the initial initiation of atorvastatin and treated with neoadjuvant chemoradiation followed by anterior resection and loop ileostomy, invasive urothelial carcinoma of the bladder treated with fulguration and intravesical Bacille Calmette-Guérin, and a history of deep venous thrombosis. His medications included apixaban 5 mg twice a day and tamsulosin 0.4 mg daily.

Additional lab work is shown in Table 1.

**Table 1:** Initial laboratory values at admission ALT: alanine transaminase, AST: aspartate transaminase, GGT: gamma-glutaryl transferase, LDH: lactate dehydrogenase, TSH: thyroid-stimulating hormone, CRP: C-reactive protein, ANA: antinuclear antibody

Lab	Value	Reference range
White blood cell count	7100/uL	4000-10,000/uL
Hemoglobin	13.1 g/dL	13-17.5 g/dL
Platelets	282,000/uL	140,000 - 400,000/uL
Creatinine	0.89 mg/dL	0.7-1.3 mg/dL
High sensitivity troponin	< 14 ng/L	< 14 ng/L
ALT	375 U/L	1-44 U/L
AST	310 U/L	14-39 U/L
Alkaline phosphatase	57 U/L	53-129 U/L
GGT	4 U/L	1-54 U/L
Aldolase	66.7 U/L	< 7.7 U/L
LDH	1090 U/L	118-225 U/L
Total bilirubin	0.7 mg/dL	0.3-1.2 mg/dL
Ferritin	146 mcg/L	22-322 mg/dL
TSH	6.59 IU/mL	0.35-5.50 IU/mL
Free T4	0.8 ng/dL	0.9-1.8 ng/dL
Sedimentation rate	70 mm/hr	0-20 mm/hr
CRP	4.9 mg/dL	0.0-1.0 mg/dL
Hepatitis A antibody	negative	negative
Hepatitis B surface antigen	negative	negative
Hepatitis B core antibody	negative	negative
Hepatitis C antibody	negative	negative
Human immunodeficiency virus antibody	nonreactive	nonreactive
ANA	negative	negative
F-Actin (anti-smooth muscle) IgG	< 20 U	< 20 U
Alpha 1 antitrypsin antibody	117 mg/dl	100-190 mg/dl
Anti-mitochondrial M2 antibody	< 20 U	< 20 U

Alanine transaminase (ALT) and aspartate transaminase (AST), aldolase, and lactate dehydrogenase (LDH) were elevated. thyroid-stimulating hormone was normal, but free T4 was low. Sedimentation rate and C-reactive protein elevations suggested an inflammatory process. Qualitative urinalysis revealed a moderate amount of blood, but only 0-2 red blood cells per high power field were seen. Urine toxicology did not detect illicit drugs, including amphetamines, cocaine, cannabinoids, barbiturates, opioids, phencyclidine, and methyl​enedioxy​methamphetamine. He was started on intravenous fluids and levothyroxine. Despite aggressive fluid resuscitation with over 9 L (fluids received at a rate up to 250 mL/hour) of normal saline over three days, CK levels rose to a peak of 18,661 U/L.

**Figure 1:**
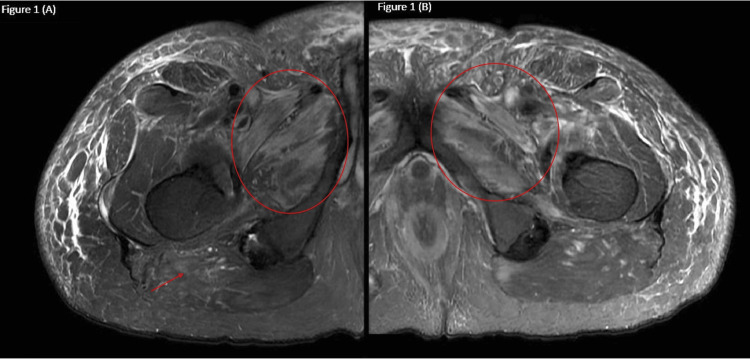
T2 weighted fast relaxation, fast spin echo sequence axial images of the right femur (A) and left femur (B). Right femur: mild diffuse interstitial edema about the posterior aspect of the thigh, moderate intramuscular edema involving the left adductor brevis, adductor magnus, and obturator externus muscles at the level of the upper thigh (circle) with fatty atrophy involving the medial and posterior thigh compartment musculature (arrow). Left femur: moderate diffuse intramuscular edema with scattered areas of fatty atrophy involving the medial and posterior thigh compartment musculature (circle).

As seen in Figure 1, an MRI of the quadriceps demonstrated edema involving the left adductor musculature and posterior and medial compartments of the thigh, concerning inflammatory myositis. A muscle biopsy was obtained.

**Figure 2:**
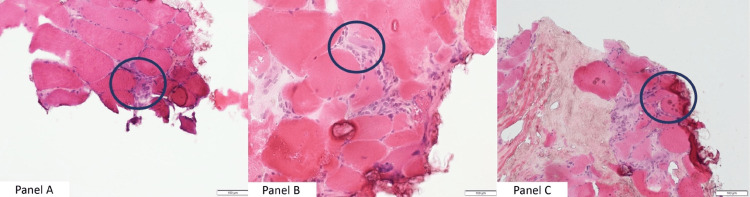
Small specimen of skeletal muscle showing polygonal and rounded fibers with increased variability in fiber size (panel B). Internal nuclei are found in about 30% of fibers. Endomysial connective tissue is moderately increased. There are slight interstitial mononuclear inflammatory cell infiltrates (panel A). Step sections of frozen tissue showed few areas of interstitial mononuclear inflammation. Hematoxylin and eosin stain. 20X (panels A, B, C).

As shown in Figure 2, histology was consistent with marked chronic active myopathy with a moderate amount of interstitial fibrosis, 5% fibers with fat replacement, and endomysial inflammation.

As shown in Table 2, immune-mediated myositis antibody testing resulted in strongly positive anti-hydroxy-3-methylglutaryl-CoA reductase (anti-HMGCR) antibodies but was negative for anti-signal recognition protein and myositis-specific and associated antibodies.

**Table 2:** Antibody panel

	Reference value	Patient’s results	MSA*	MAA**
HMGCR antibody, IgG	0-19 units	>200 units		
SAE1 (SUMO activating enzyme) antibody	Negative	Negative	X	
NXP2 (Nuclear matrix protein-2) antibody	Negative	Negative	X	
MDA5 (CADM-140) antibody	Negative	Negative	X	
TIF-1 gamma (155 kDa) antibody	Negative	Negative	X	
Mi-2 (nuclear helicase protein) antibody	Negative	Negative	X	
P155/140 antibody	Negative	Negative	X	
PL-12 (alanyl-tRNA synthetase) antibody	Negative	Negative	X	
PL-7 (threonyl-tRNA synthetase) antibody	Negative	Negative	X	
OJ (isoleucyl-tRNA synthetase) antibody	Negative	Negative	X	
EJ (glycyl-tRNA synthetase)	Negative	Negative	X	
SRP (signal recognition particle) antibody	Negative	Negative	X	
Jo-1 (histidyl-tRNA synthetase) antibody, IgG	0-40 AU/mL	0 AU/mL	X	
Ku antibody	Negative	Negative		X
Smith/RNP (ENA) antibody, IgG	0-19 Units	4 Units		X
PM/Scl 100 antibody, IgG	Negative	Negative		X
SSA-52 (Ro52) extractable nuclear antigen (ENA) antibody	0-40 AU/mL	0 AU/mL		X
SSA-60 (Ro60) extractable nuclear antigen (ENA) antibody, IgG	0-40 AU/mL	1 AU/mL		X
Fibrillarin (U3 RNP) antibody, IgG	Negative	Negative		X

The patient was started on 40 mg of prednisone daily and methotrexate 15 mg per week with folic acid supplementation. At one month follow-up, CK levels decreased to 6078 U/L, AST to 180 U/L, ALT to 293 U/L, and LDH to 724 U/L. Clinically, muscle weakness improved and myalgia resolved. Glucocorticoids were slowly tapered, and methotrexate was increased to 20 mg weekly with continued clinical improvement, including the normalization of muscle strength in all extremities, normalization of gait, and a decrease in CK.

## Discussion

We present a case of chronic anti-HMGCR positive immune-mediated myositis. Our case highlights a rare autoimmune condition related to prior statin exposure with a delay in diagnosis. Our case is unique in demonstrating the subacute but ongoing chronicity in our patient, both in symptoms, as well as in histologic changes seen on muscle biopsy.

While statins generally have an acceptable benefit-to-risk profile, 5-20% of patients [1] stop the medication due to muscle-related adverse effects, including muscle cramping, nonspecific myalgia, and elevations in CK, among others [2]. Risk factors for adverse events related to statins include statins metabolized CYP3A4, increased dosage, comorbid neuromuscular diseases such as amyotrophic lateral sclerosis, genetic mutations such as SLCO1B1 gene, hypothyroidism, liver or kidney disease, low vitamin D levels, and the use of other medications that inhibit CYP3A4, among others [4]. Rarely, statin-induced immune-mediated necrotizing myositis occurs and may pose a diagnostic challenge. While some cases resolve with cessation of statins, many cases develop ongoing persistent necrotizing myositis. A chronic phenotype, mimicking limb-girdle muscular dystrophy, has been described, in particular in children [3]. Our case accentuates this chronic syndrome in an older adult. Patients who have the longstanding disease may histologically demonstrate muscle fiber replacement with adipose tissue and fibrosis [5]. Our biopsy is consistent with chronic disease, demonstrating both.

Some forms of immune-mediated necrotizing myositis are associated with malignancy [6]. Epidemiologically, anti-HMGCR myopathy correlates weakly with adenocarcinoma of the gastrointestinal tract, esophagus [7], breasts, uterus, ovaries, lymphoma, and melanoma [6,8,9]. However, one study did not find a correlation with malignancy [10]. Our patient had a prior history of both rectal adenocarcinoma and urothelial carcinoma, both of which developed following his initial statin exposure. Unfortunately, while he was symptomatic prior to the malignancy, antibodies for anti-HMG-CoA reductase were not checked at that time or during the onset of his malignancies. This does limit our ability to correlate his malignancy and muscle symptoms; however, an association is possible. Further chemotherapy was not indicated.

Cases of anti-HMGCR antibody-positive myositis have been reported without statin exposure, particularly in younger patients, and may be linked to natural sources of statins in the environment, e.g., mushrooms. The mean age of patients with anti-HMGCR myopathy not triggered by statins tends to be younger (40 years of age). These cases also tend to be more severe and less responsive to treatment [10].

There are no large, double-blinded, placebo-controlled clinical trials to guide treatment, but case series do support options for treatments. Typically, treatment involves discontinuation of statins and administration of glucocorticoids [11], usually in combination with immunosuppressive medications such as methotrexate, azathioprine, mycophenolate mofetil, tacrolimus, or cyclosporine [12,13]. It is important to start immunosuppressive therapy early at diagnosis as discontinuation of statins alone usually is not sufficient to improve muscle weakness. Refractory cases may require intravenous immunoglobulin, rituximab, or cyclophosphamide [11]. Intravenous immunoglobulin monotherapy at a dose of 2 g/kg of body weight given monthly was shown to improve muscle strength in a small cohort of three subjects who declined glucocorticoid medications [11]. Clinical experience suggests that combination therapy may be most efficacious. Clinical examination, functional testing, and monitoring of CK levels may help determine therapeutic response.

In summary, we present a rare case of chronic anti-HMGCR positive immune-mediated necrotizing myopathy. Given our patient’s distant temporal use of statin, the diagnosis was challenging. However, appropriate clinical suspicion and antibody testing were able to seal the diagnosis.

## Conclusions

We highlight a case of undiagnosed necrotizing myositis in a male three years after statin exposure and cessation. Statins are commonly prescribed and may be discontinued at the onset of muscle symptoms. However, symptoms may persist and may not completely resolve with medication cessation alone. Diagnosis may be missed altogether because of the rarity of this condition, and symptoms may be wrongly attributed to other causes. Suspicion should prompt withdrawal of the offending medicine and checking for CK levels. Diagnosis with serology and imaging helps confirm diagnosis and early treatment with glucocorticoids, and immunosuppressant medications may prevent irreversible muscular damage. Improvement in CK levels and symptoms points toward the efficacy of treatment. Although rare, this condition should be considered in patients who have continued symptoms and elevated CK levels even after drug cessation.
